# Role of iPSC-derived pericytes on barrier function of iPSC-derived brain microvascular endothelial cells in 2D and 3D

**DOI:** 10.1186/s12987-019-0136-7

**Published:** 2019-06-06

**Authors:** John J. Jamieson, Raleigh M. Linville, Yuan Yuan Ding, Sharon Gerecht, Peter C. Searson

**Affiliations:** 1 Department of Chemical and Biomolecular Engineering, Johns Hopkins University, 3400 North Charles Street, Baltimore, MD 21218 USA; 2 Institute for Nanobiotechnology, 100 Croft Hall, Johns Hopkins University, 3400 North Charles Street, Baltimore, MD 21218 USA; 3 Department of Biomedical Engineering, Johns Hopkins University, 720 Rutland Avenue, Baltimore, MD 21205 USA; 4 Department of Materials Science and Engineering, Johns Hopkins University, 3400 North Charles Street, Baltimore, MD 21218 USA

**Keywords:** Blood–brain barrier, Brain microvascular endothelial cells, Pericytes, Induced pluripotent stem cells, Tissue engineering, Transendothelial electrical resistance

## Abstract

**Background:**

Pericytes of the blood–brain barrier (BBB) are embedded within basement membrane between brain microvascular endothelial cells (BMECs) and astrocyte end-feet. Despite the direct cell–cell contact observed in vivo, most in vitro BBB models introduce an artificial membrane that separates pericytes from BMECs. In this study, we investigated the effects of pericytes on BMEC barrier function across a range of in vitro platforms with varied spatial orientations and levels of cell–cell contact.

**Methods:**

We differentiated RFP-pericytes and GFP-BMECs from hiPSCs and monitored transendothelial electrical resistance (TEER) across BMECs on transwell inserts while pericytes were either directly co-cultured on the membrane, indirectly co-cultured in the basolateral chamber, or embedded in a collagen I gel formed on the transwell membrane. We then incorporated pericytes into a tissue-engineered microvessel model of the BBB and measured pericyte motility and microvessel permeability.

**Results:**

We found that BMEC monolayers did not require co-culture with pericytes to achieve physiological TEER values (> 1500 Ω cm^2^). However, under stressed conditions where TEER values for BMEC monolayers were reduced, indirectly co-cultured hiPSC-derived pericytes restored optimal TEER. Conversely, directly co-cultured pericytes resulted in a decrease in TEER by interfering with BMEC monolayer continuity. In the microvessel model, we observed direct pericyte-BMEC contact, abluminal pericyte localization, and physiologically-low Lucifer yellow permeability comparable to that of BMEC microvessels. In addition, pericyte motility decreased during the first 48 h of co-culture, suggesting progression towards pericyte stabilization.

**Conclusions:**

We demonstrated that monocultured BMECs do not require co-culture to achieve physiological TEER, but that suboptimal TEER in stressed monolayers can be increased through co-culture with hiPSC-derived pericytes or conditioned media. We also developed the first BBB microvessel model using exclusively hiPSC-derived BMECs and pericytes, which could be used to examine vascular dysfunction in the human CNS.

**Electronic supplementary material:**

The online version of this article (10.1186/s12987-019-0136-7) contains supplementary material, which is available to authorized users.

## Background

Brain microvascular endothelial cells (BMECs) in capillaries are surrounded by astrocyte end-feet [1, 2], with pericytes and basement membrane located between these two cell layers [3–8]. The density of pericytes along the vasculature varies greatly across tissues, as high as 1 pericyte per 3–5 ECs in the brain and as low as 1 pericyte per 10–100 ECs in skeletal muscle [9, 10]. Despite their intimate association with BMECs, pericytes are the least studied of the cellular components of the blood–brain barrier (BBB).

Pericytes are known to play an important role in the formation of the cerebrovasculature during development [11, 12] and in response to trauma [13, 14], however, the role of pericytes in BBB function is less well established. Pericyte-deficient mice show BMEC abnormalities including increased permeability to water and tracers, increased transcytosis, upregulation of leukocyte adhesion molecules, and abnormal tight junction morphology [15, 16]. However, most BBB markers in BMECs are unaffected by pericyte deficiency [16] and the overall expression of tight junction proteins remains unchanged [15, 16], although decreases in ZO-1 and occludin expression are observed during aging [17].

Other evidence for the role of pericytes in BBB function comes from in vitro transwell experiments where the presence of pericytes in the basolateral chamber increases transendothelial electrical resistance (TEER) [16, 18–20]. However, many of these experiments were performed with BMECs that had TEER values well below the range considered to be physiological (1500–8000 Ω cm^2^) [20–24]. For example, the TEER of primary murine BMECs increased from about 35 Ω cm^2^ to about 140 Ω cm^2^ with pericytes in the basolateral chamber [16]. In addition, these studies do not recapitulate the direct cell–cell contact observed in vivo.

To address these limitations, we have differentiated pericytes and brain microvascular endothelial cells from human induced pluripotent cells (hiPSCs), and assessed the influence of derived pericytes (dhPCs) on the paracellular barrier function of derived brain microvascular endothelial cells (dhBMECs) in three different spatial arrangements. First, we cultured dhBMECs on the apical side of a transwell support with dhPCs in the basolateral chamber in a standard non-contact configuration and measured TEER values. We also examined direct co-culture on the apical side of the chamber in three conditions: dhPCs seeded on dhBMECs, dhBMECs seeded on dhPCs, and simultaneous seeding of dhBMECs and dhPCs. Second, to allow pericyte migration in 3D, we formed dhBMEC monolayers on gels seeded with dhPCs on a transwell support. Finally, to recapitulate the spatial arrangement of pericytes in the brain, we co-cultured dhPCs and dhBMECs in three-dimensional microvessels under shear flow. Using these configurations, we provide insight into the role of pericytes on barrier function of monolayers of dhBMECs.

## Methods

### dhBMEC differentiation

BC1 [25] and BC1-GFP [26] hiPSC lines were maintained and differentiated to dhBMEC as previously described [27, 28] with minor modifications. All materials were purchased from Thermo Fisher Scientific unless otherwise specified. Briefly, hiPSCs were cultured feeder-free on tissue culture treated plates (Cell Star) coated with vitronectin and maintained in E8 medium replaced daily. hiPSCs were passaged approximately every 4 days by dissociation with 0.5 mM EDTA (Promega), centrifugation, and reseeded with 10 µM of the ROCK inhibitor Y-27632 (STEMCELL Technologies) for the first 24 h. At 30–50% confluence, differentiation was initiated by switching to differentiation medium (DMEM/F12 supplemented with 20% KOSR, 1% non-essential amino acids, 0.5% GlutaMAX, and 0.8 μM beta-mercaptoethanol). Medium was changed daily through day 5 of differentiation. On day 6, the cells were switched to dhBMEC medium for 2 days. The dhBMEC medium consisted of endothelial cell serum-free medium supplemented with 1% platelet-poor plasma-derived human serum (Sigma), 2 ng mL^−1^ bFGF (R&D Systems), and 10 μM all-trans retinoic acid (Sigma). On day 8, cells were dissociated with accutase for 15–20 min and sub-cultured onto glass (5 × 10^5^ cells cm^−2^) or transwells (3 × 10^6^ cells cm^−2^) in dhBMEC medium with 10 μM ROCK inhibitor Y-27632. After 24 h, the medium was switched to dhBMEC medium. In some experiments, cells were subcultured in dhBMEC medium alone (no ROCK inhibitor), resulting in confluent dhBMEC monolayers with suboptimal TEER values. Glass dishes and transwell membranes were coated overnight with 50 µg mL^−1^ human collagen IV (Sigma) and 25 µg mL^−1^ human fibronectin (Sigma).

### dhPC differentiation

BC1 and C12-RFP hiPSC lines were maintained and differentiated to dhPCs as previously described with minor modifications [29]. hiPSCs were cultured on a feeder layer of mouse embryonic fibroblasts (MTI Globalstem) on tissue culture treated plates (Cell Star) coated with gelatin (Sigma). To initiate differentiation, hiPSC were dissociated with 0.5 mM EDTA, strained through a 40 µm mesh (BD Falcon), and seeded on collagen IV (Corning)-coated plates. Cells were cultured for 6 days in a differentiation medium composed of MEM α, 10% FBS (Hyclone), and 0.1 mM β-mercaptoethanol replaced daily. On day 6, cells were dissociated with TrypLE Express, strained through a 40 µm mesh, seeded on collagen IV-coated plates, and grown in endothelial cell growth medium (PromoCell) with the addition of 10 μM SB431542 (Tocris) to promote early vascular cell (EVC) specification through TGF-β inhibition, and 50 ng mL^−1^ VEGF (R&D Systems) to promote EVC proliferation. The medium was changed every other day. On day 12, EVCs were dissociated with TryPLE Express and re-plated on uncoated tissue culture-treated six-well plates in DMEM (Gibco 11965) with 10% FBS (Gibco 10082), conditions which favor the selection and enrichment of pericyte-like cells. The medium was replaced every other day for the next 6 days. dhPCs were further expanded in Pericyte Medium (Sciencell) and used between passages 1–4. Experiments involving dhPC co-culture or conditioned medium utilized dhBMEC medium, as opposed to pericyte medium or a blend of the two, as dhBMEC did not maintain barrier function in pericyte medium (data not shown).

Primary human brain vascular pericytes (hBVP, Sciencell) were cultured in Pericyte Medium (Sciencell) and used between passages 2–5. Primary human placental pericytes (Promocell) were cultured in Pericyte Growth Medium (Promocell) and used between passages 2–5.

### Immunocytochemistry

**Fig. 1:**
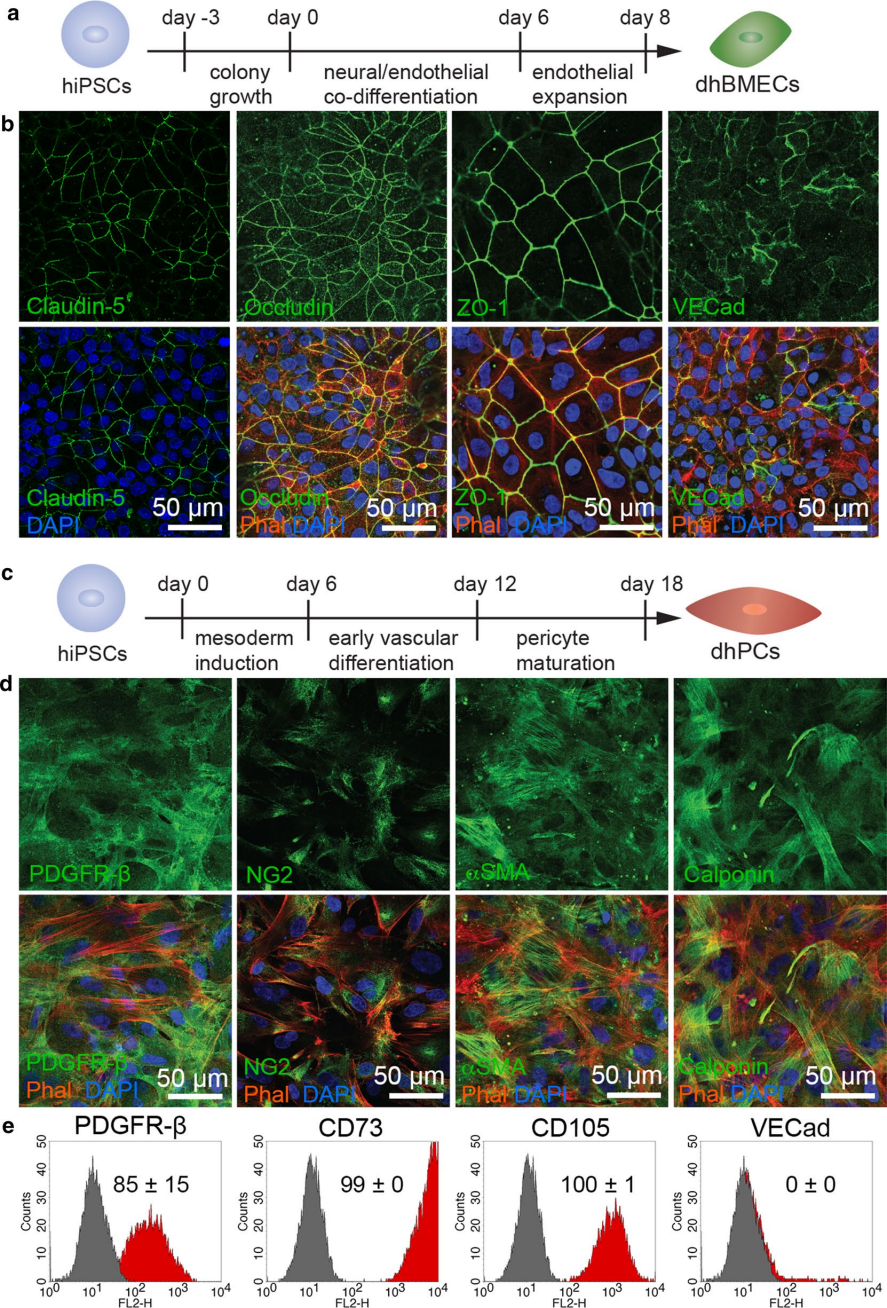
Differentiation and characterization of dhBMECs and dhPCs. **a** Differentiation scheme for dhBMECs. **b** Immunofluorescence staining of dhBMEC monolayers for proteins associated with tight (claudin-5, occludin, and ZO-1) and adherens (VECad) junctions, performed 48 h after dhBMEC subculture, displayed alone for clarity (top row), and with cell nuclei and f-actin labeled by DAPI and phalloidin, respectively (bottom row). **c** Differentiation scheme for dhPCs. **d** Immunofluorescence staining of dhPCs for established pericyte and mural cell markers (PDGFRβ, NG2, αSMA, and calponin) displayed alone for clarity (top row), and with cell nuclei and f-actin labeled by DAPI and phalloidin, respectively (bottom row). **e** Representative live-cell flow cytometry histograms of dhPCs for pericyte and mesenchymal surface markers (PDGFRβ, CD73, CD105, and absence of VECad). The percentages listed on each histogram are the mean ± SD of at least three biological replicates

Immunostaining was performed as previously described [29]. Cells were fixed in 3.7% paraformaldehyde (Sigma) and permeabilized with 0.1% Triton-X (Sigma). For select immunostains (Additional file 1: Figs. S2B, S4B, and claudin-5 in Fig. 1b) 10 min of ice cold methanol (Sigma) was used as an alternative fixation technique. Cells were blocked in 1% bovine serum albumin (Sigma) for 1 h, incubated with primary antibodies overnight at 4 °C, and incubated with secondary antibodies for 1 h at room temperature, rinsing with DPBS three times between each step. See Additional file 1: Table S1 for details of antibodies used. Monolayers were imaged on a Zeiss LSM 780 or Zeiss LSM 800 using ZEN Black or ZEN Blue software, or imaged on a Nikon TiE Confocal microscope with NIS Elements software.

### Flow cytometry

Cells were collected using TrypLE Express and resuspended in 0.1% bovine serum albumin (Sigma). Cells were incubated with conjugated antibodies (Additional file 1: Table S1) on ice in the dark for 45 min and washed three times with 0.1% bovine serum albumin. Marker expression was measured by a BD FACScaliber cytometer. Forward-side scatter plots were used to exclude dead cells. All analyses were done using corresponding isotype controls.

### Transendothelial electrical resistance (TEER)

TEER was measured daily for 1 week following cell seeding on 24-well Transwell inserts with a 0.4 µm pore size (Corning 3470), as previously described [28]. An EVOM2 system (World Precision Instruments) with a STX2 probe was used to measure total resistance (Ω). All TEER values were normalized to the area of the membrane (0.33 cm^2^) and corrected for the resistance without cells. All TEER experiments were performed with at least 2 duplicate wells, and at least three independent differentiations. For a given biological replicate, the peak TEER represents the TEER value on whichever day the average of the technical replicates for that condition yielded the maximum TEER value. For plots of TEER versus time, TEER values were normalized to the peak value of the control (no dhPCs), such that each control biological replicate reaches a maximum relative TEER of 1.0 at its highest value.

### Real-time quantitative RT-PCR

Two-step RT-PCR was performed as previously described on direct contact co-cultures of dhPC and dhBMEC [30]. Total RNA was extracted with TRIzol (Gibco, Invitrogen), and purified using the DirectZol RNA purification kit. Reverse transcriptase MMLV (Promega Co., Madison, WI) and oligo (dT) primers (Promega) were used to generate cDNA, as per the manufacturer’s instructions. Gene expression was measured using a StepOne Real-Time PCR System (Applied Biosystems) with TaqMan Universal PCR Master Mix and the following Gene Expression Assays (Applied Biosystems): CLDN5 (Claudin-5, Hs00533949_s1); OCLN (Occludin, Hs00170162_m1). Relative gene expression was normalized to GAPDH by using the standard curve method. For each primer set, the comparative cycle threshold (∆∆Ct) was used to calculate the amplification differences between the different samples.

### 2.5D gel co-cultures

Collagen I gels were formed on transwell inserts either with or without embedded dhPCs by adapting previous protocols [27]. Rat tail collagen I (Corning) was diluted in M199 to achieve a final concentration of 2.5 mg mL^−1^. 0.2 M NaOH was added in 1 µL aliquots while mixing on ice until a pH of 7.5 was obtained. 56 µL of solution was pipetted onto each transwell membrane and incubated for 30 min at 37 °C to allow gel formation. For assessing dhPC migration in response to dhBMECs, 200 µL gels with or without embedded dhPCs were formed in 96 well plates. Post-gelation, dhPC viability was verified by calcein/propidium iodide live/dead staining (Thermo Fisher) according to manufacturer instructions. dhBMEC medium containing 50 µg mL^−1^ collagen IV and 25 µg mL^−1^ fibronectin was added on top of the gel and incubated overnight prior to dhBMEC seeding. Pericyte position was defined relative to the bottom of the well.

### Microvessel fabrication, permeability, and cell tracking

Brain microvessels were fabricated as previously reported [27, 31] with minor modifications. Briefly, a 150 µm cylindrical template rod is embedded within a 7 mg mL^−1^ collagen I gel and then removed to leave a hollow channel. dhPCs were suspended at 6 × 10^6^ cells mL^−1^ and seeded into the channel. After a 1-h attachment period, dhBMEC were seeded at a density of 8 × 10^7^ cells mL^−1^. Microvessels were perfused with the same medium as used in other configurations. Live-cell imaging was conducted on day 2 after seeding using an inverted microscope (Nikon Eclipse Ti-E) maintained at 37 °C and 5% CO_2_. Lucifer yellow and 10 kDa dextran permeability was calculated as previously reported [31]. Imaris 8 was used for cell tracking experiments.

### Statistical analysis

GraphPad Prism 7 and IGOR Pro 6 were used for statistical analysis. Student’s t-test was employed for comparisons between two conditions, while ANOVA with multiple comparisons was used for tests with three or more conditions. P-values were multiplicity adjusted using either the Dunnett or Tukey multiple comparisons tests, as appropriate. Differences were considered statistically significant for P < 0.05, with the following thresholds: *P < 0.05, **P < 0.01, ***P < 0.001.

## Results

### Differentiation and characterization of dhBMECs and dhPCs

Brain microvascular endothelial cells (dhBMECs) were obtained by differentiation from the BC1 hiPSC line [32]. The protocol for differentiation of dhBMECs has been reported previously [33] (Fig. 1a). dhBMEC monolayers express a wide range of BBB markers, including tight junction (TJ) proteins (Fig. 1b), transporters, and efflux pumps, and typically attain transendothelial electrical resistance (TEER) > 1500 Ω cm^2^ [27, 28, 31, 34–37]. In addition, microvessels formed by seeding dhBMECs within channels patterned in type I collagen display physiological barrier function [31].

Pericyte-like cells (dhPCs) were differentiated from the BC1 or C12 hiPSC lines using a previously published protocol [29] (Fig. 1c). This differentiation begins with mesoderm induction followed by early vascular specification, which yields a mixture of endothelial and pericyte-like cells. Pericyte-like cells are purified from this mixture by subculture onto uncoated cultureware by preferential attachment. After an additional 6 days of maturation in pericyte medium, cells were fixed and stained for established pericyte and mural cell markers including PDGFRβ, NG2, αSMA, and calponin (Fig. 1d). Flow cytometry demonstrated positive expression for a panel of pericyte and mesenchymal surface markers including PDGFRβ, CD73, and CD105. In vivo, αSMA and calponin expression are restricted to mural cells along brain arterioles and arteries [38], however, these markers are often upregulated during culture [39], complicating identification of pericytes. As a result, dhPCs are denoted as pericyte-like cells. Notably, dhPCs were negative for VECad expression, indicating the absence of endothelial cells (Fig. 1e).

To assess the suitability of dhPCs for this study, we compared expression of established pericyte markers in dhPCs to human brain vascular pericytes (hBVPs) and human placental pericytes (hPPs). Immunofluorescence images of hBVPs revealed comparable expression of PDGFRβ, NG2, αSMA, and calponin to dhPCs (Fig. 1d and Additional file 1: Fig. S1A). From flow cytometry analysis, all three pericyte populations exhibited comparable expression of the pericyte and mesenchymal surface markers PDGFRβ, CD73, and CD105 (Additional file 1: Fig. S1B). However, approximately 17% of hBVPs exhibited elevated VECad expression, which was negligible in the other pericyte populations (Additional file 1: Fig. S1C). CD31 immunofluorescence confirmed the presence of a subset of endothelial-like cells within the hBVP population, suggesting impurities in the commercial isolation or trans-differentiation (Additional file 1: Fig. S1D). From these comparisons, we concluded that dhPCs were comparable to primary brain pericytes in the expression of multiple established pericyte biomarkers and have a higher fraction of cells with the specified biomarkers.

Recapitulating the spatial organization of dhPCs and dhBMECs in vitro is difficult and hence we assessed 3 configurations with increasing complexity: (1) 2D culture in transwells (either non-contact or with direct contact), (2) 2.5D culture with dhBMEC monolayers formed on a hydrogel with or without embedded pericytes on a transwell membrane, and (3) co-culture of dhPCs in tissue-engineered dhBMEC microvessels.

### Non-contact culture of dhPCs and dhBMECs in transwells

**Fig. 2:**
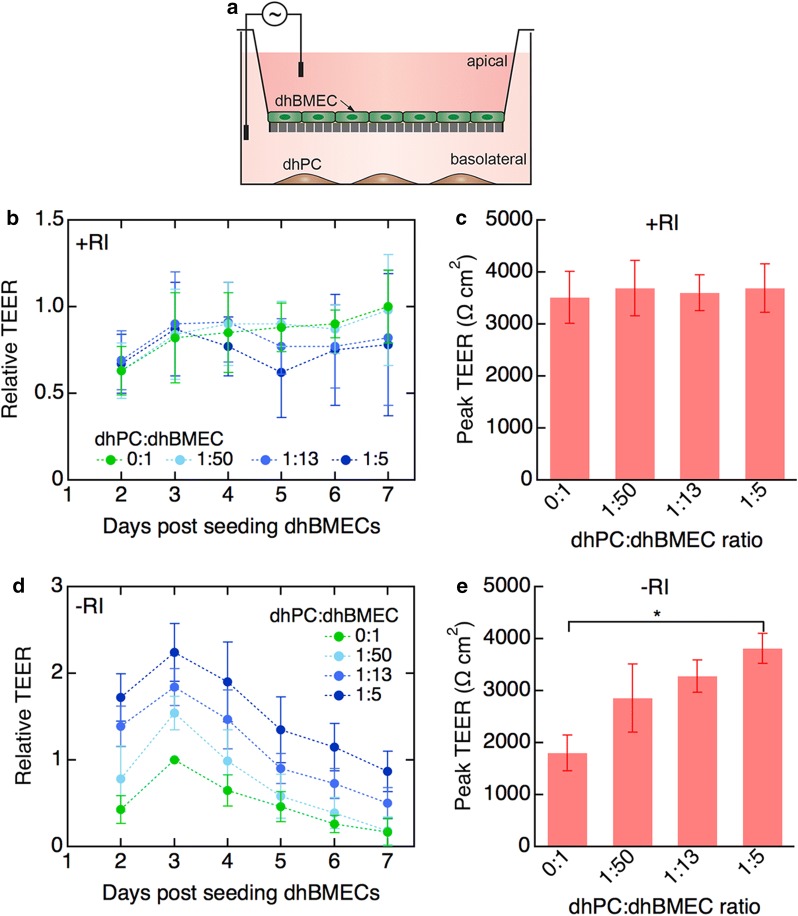
Barrier function of dhBMEC monolayers on transwells in non-contact co-culture with dhPCs. **a** Schematic illustration of indirect co-culture in a transwell device. **b** Time dependence of TEER values for dhBMEC monolayers with different dhPC concentrations in the basolateral chamber. TEER values were normalized to the peak value of the control (no dhPCs), such that each biological replicate of the control reaches a maximum relative TEER of 1.0 at its highest point. **c** Peak TEER for dhBMEC monolayers. Peak TEER represents the TEER on whichever day the average of the technical replicates for that condition yielded the maximum TEER value. **d** Time dependence of TEER values for stressed dhBMEC monolayers (no ROCK inhibitor (-RI) during subculture) with different dhPC concentrations in the basolateral chamber. **e** Peak TEER for stressed dhBMEC monolayers. Data represent mean ± SEM, *P < 0.05. All experiments were performed with three biological replicates (independent dhBMEC differentiations) and at least two technical replicates (transwell experiments for each differentiation)

To assess the role of dhPCs on the barrier function of dhBMECs in 2D, we cultured pericytes in the basolateral chamber of a transwell such that there was no contact between the two cell types (Fig. 2a). Under optimal conditions with ROCK inhibitor added during seeding, monocultured dhBMECs maintained high TEER from 2 to 7 days post-seeding, and the addition of dhPCs to the basolateral chamber at dhPC:dhBMEC ratios of 1:50, 1:13, and 1:5 did not alter TEER values, although there was a slight, but not significant, decrease for higher dhPC:dhBMEC ratios after 5–7 days, possibly due to nutrient competition (Fig. 2b). The peak TEER achieved under each condition was approximately 3500 Ω cm^2^, regardless of dhPC concentration (Fig. 2c). ROCK inhibitor is widely used during passaging of hiPSCs and, in some cases, during dhBMEC seeding, to alleviate cell stress [40, 41]. ROCK inhibitor improved cell attachment and spreading, promoting rapid and continuous monolayer formation (Additional file 1: Fig. S2A), but did not affect the expression or localization of TJ proteins (Additional file 1: Fig. S2B), in agreement with previous data [40]. Note that dhPCs were not exposed to ROCK inhibitor, as the compound was removed on day 1 just prior to co-culture initiation.

Having established that non-contact co-culture of pericytes had no influence on TEER values of dhBMEC monolayers under normal culture conditions, we assessed the role of pericytes on stressed monolayers. We define stressed monolayers as monolayers formed in the absence of ROCK inhibitor with reduced peak TEER values of around 1800 Ω cm^2^ which progressively declined over 3 days post-seeding (Fig. 2d, e). Co-culture with dhPCs in the basolateral chamber increased TEER throughout the 7-day culture period (Fig. 2d). Peak TEER increased with increasing pericyte concentration, and was statistically higher than the control (no dhPCs) at the 1:5 dhPC:dhBMEC ratio. At this ratio, the TEER was 3800 Ω cm^2^, matching values observed in unstressed dhBMEC monolayers (Fig. 2e).

We then assessed whether the effect of dhPCs in increasing the TEER of stressed dhBMEC monolayers was exerted during or after the formation of the confluent monolayer. For dhBMEC monolayers co-cultured with dhPCs in the basolateral chamber starting on day 1 (CC d1), as compared with co-culture starting on day 0 (CC d0), a similar concentration-dependent increase in TEER was observed, indicating that dhPCs do not need to be present during dhBMEC seeding in order to increase TEER by day 2 (Additional file 1: Fig. S3). However, significant increases were noted for d0-initiated co-cultures compared to d1-initiated co-cultures, suggesting that the increase in TEER is related to the total time in co-culture. Lastly, we assessed whether the TEER increases observed in non-contact co-culture of stressed monolayers could be replicated using conditioned medium. We found that dhPC-conditioned medium increased TEER to a similar extent as medium and high concentrations of dhPCs (Additional file 1: Fig. S3). We compared the effect of dhPC-conditioned medium to that of medium conditioned by primary brain pericytes and found a similar influence on TEER (Additional file 1: Fig. S4A). Claudin-5 and occludin expression and localization appeared consistent across media conditions (Additional file 1: Fig. S4B).

Taken together, these results suggest that dhPCs are not necessary to achieve physiological barrier function in dhBMEC monolayers, but that dhPCs or dhPC-conditioned medium are capable of improving the barrier function of stressed dhBMEC monolayers through the expression of soluble factors. These results are summarized in Table 1.

**Table 1:** Summary of TEER results from in vitro co-culture platforms

Platform	PC location	Condition	dhPC:dhBMEC ratio	TEER ± SEM (Ω cm^2^)	Comments
2D transwell	Basolateral PCs	Unstressed ECs	0:1	3510 ± 500	TEER independent of dhPC concentration
			1:50	3690 ± 540	
			1:13	3600 ± 350	
			1:5	3690 ± 470	
		Stressed ECs	0:1	1800 ± 350	Increase in TEER with increasing dhPC concentration
			1:50	2860 ± 660	
			1:13	3280 ± 320	
			1:5	3810 ± 290	
	Apical PCs		0:1	2680 ± 130	
		P-on-E	1:50	2300 ± 430	Adding dhPCs after dhBMEC decreased TEER
			1:5	1210 ± 450	
		E-on-P	1:50	1480 ± 790	Seeding dhPCs before dhBMEC decreased TEER
			1:5	200 ± 180	
		E + P	1:50	1490 ± 730	Co-seeding of dhPCs and dhBMEC decreased TEER
			1:5	300 ± 270	
2.5D gel transwell	Embedded PCs		0:1	2550 ± 970	Embedding dhPCs in Col I gel did not affect TEER
			1:13	2410 ± 520	
3D microvessels	Vessel-lining PCs		1:13	N/A	Incorporating dhPC did not affect permeability to LY (444 Da)

### Direct co-culture of dhPCs and dhBMECs in 2D transwells

**Fig. 3:**
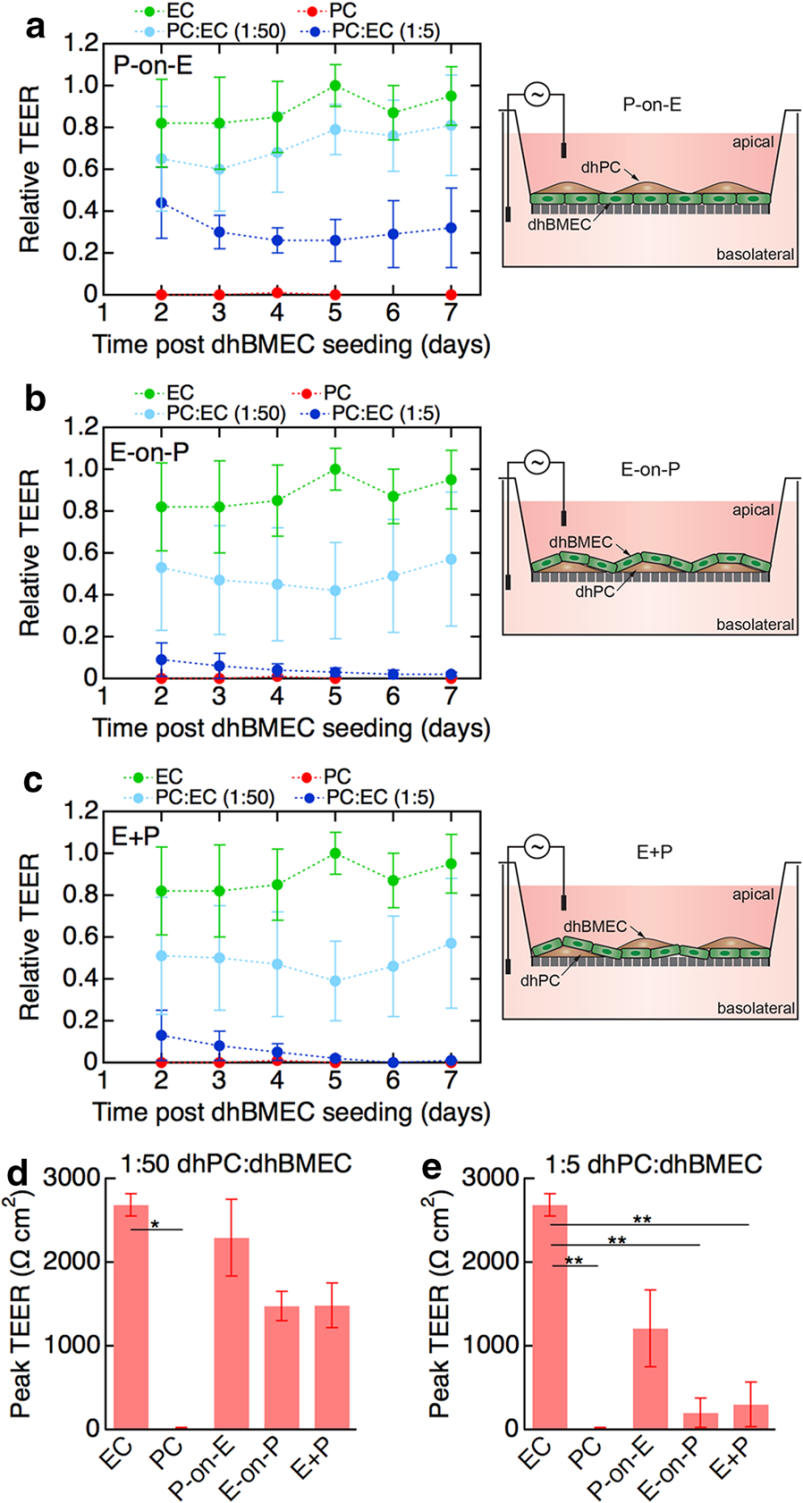
Barrier function of dhBMEC monolayers on transwells in contact co-culture with dhPCs. **a**–**c** Time dependence of TEER values for the experimental configurations shown in the schematic illustrations. **a** dhPCs on dhBMECs (P-on-E). **b** dhBMECs on dhPCs (E-on-P). **c** A mixture of dhBMECs and dhPCs. (E + P). In each configuration experiments were performed at PC:EC ratios of 1:50 (low) and 1:5 (high). TEER values are also shown for dhBMEC monolayers (EC) and dhPCs only (PC). TEER values have been normalized to the peak of the control (no dhPCs), such that each biological replicate of the control reaches a maximum relative TEER of 1.0 at its highest point. **d** Peak TEER achieved by each co-culture condition with low (1:50) PC:EC ratio. **e** Peak TEER at high (1:5) PC:EC ratio. Data represent mean ± SEM, *P < 0.05, **P < 0.01. All experiments were performed with three biological replicates (independent dhBMEC differentiations) and at least two technical replicates (transwell experiments for each differentiation)

To assess contact co-culture of dhPCs and dhBMECs in 2D, we evaluated three configurations: dhPCs seeded on dhBMEC monolayers (P-on-E), dhBMECs seeded on dhPCs (E-on-P), and a mixture of dhBMECs/dhPCs (E + P) (Fig. 3a–c). For sequential seeding, the second cell type was seeded 24 h after the first.

The addition of dhPCs to a confluent monolayer of dhBMECs resulted in a sustained decrease in TEER that was largest at the higher dhPC concentration (Fig. 3a). Seeding dhBMECs on dhPCs also resulted in a decrease in TEER values with increasing pericyte concentration (Fig. 3b). Similarly, seeding a mixture of dhBMECs/dhPCs resulted in a progressive decrease in TEER (Fig. 3c). Although the average peak TEER values for seeding dhBMECs on dhPCs and dhBMEC/dhPC mixtures at the lower dhPC concentration (1:50 ratio) were substantially lower than control values (no dhPCs), the difference was not significant (Fig. 3d). At the higher dhPC concentration (1:5 ratio), culture of dhPCs on a dhBMEC monolayer did not result in a statistically significant decrease in TEER (Fig. 3e). However, seeding dhBMECs on dhPCs or dhBMEC/dhPC mixtures resulted in a significant decrease in TEER (Fig. 3e). In general, allowing dhBMECs to form a monolayer prior to seeding dhPCs was the least disruptive condition. Seeding dhPCs first or concurrently with dhBMECs was most disruptive, implying that pericytes prevented the formation of a confluent monolayer of dhBMECs and the formation of a continuous tight junction network. Compared to non-contact culture, which had no effect on TEER of an unstressed monolayer, direct contact co-culture for most conditions resulted in barrier disruption.

### Confocal imaging of 2D direct co-culture of dhPCs and dhBMECs

**Fig. 4:**
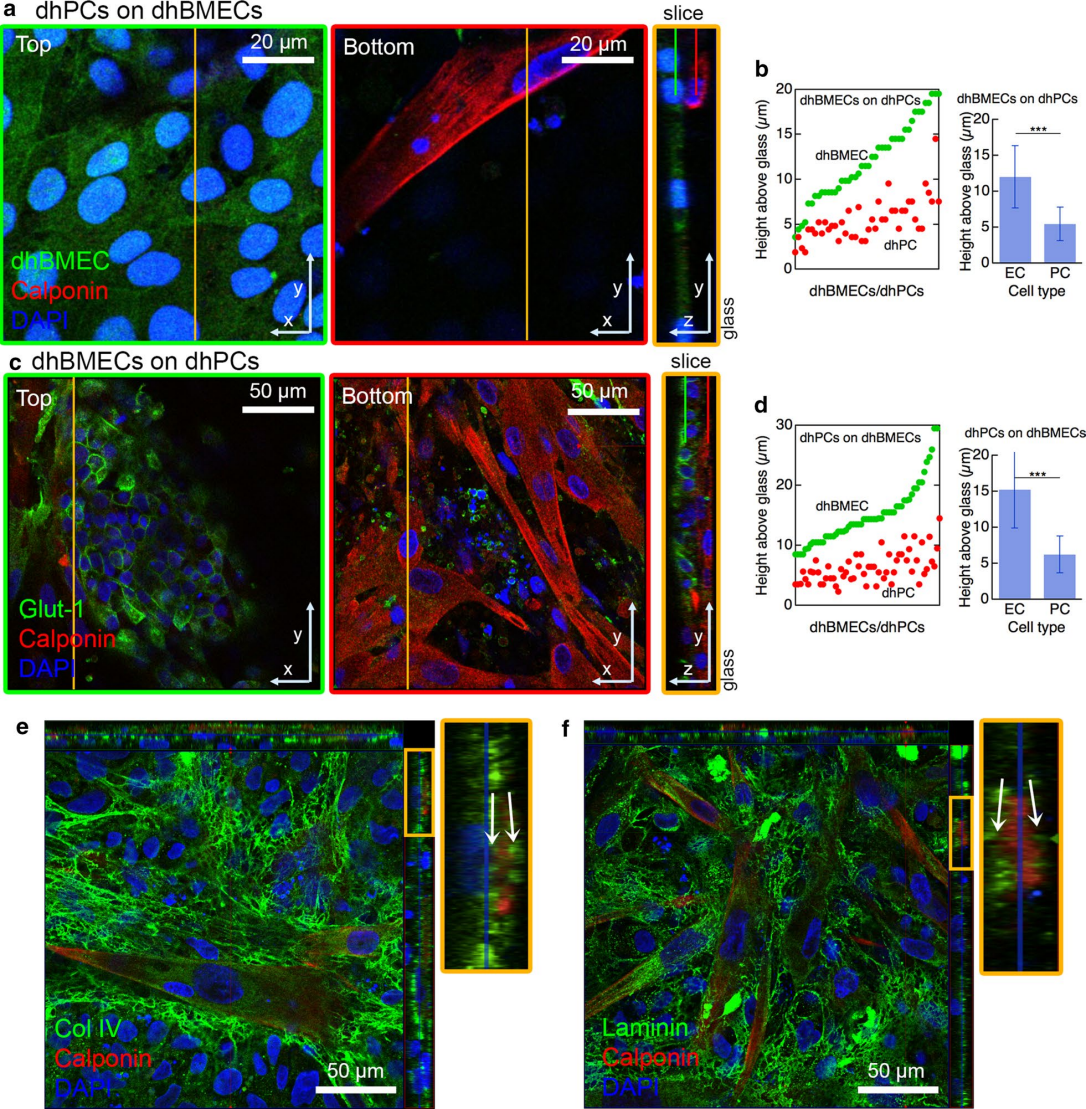
Confocal images of direct contact 2D co-culture of dhPCs and dhBMECs. **a**, **c** Confocal z-stack and cross-section images 7 days after seeding dhPCs on dhBMECs (**a**) and dhBMECs on dhPCs (**c**). In **a** and **c** XY slices are shown at the height of the dhBMEC layer (top), and at the height of the dhPC layer (bottom). The orange lines denote the plane of the YZ cross section. The green and red lines segments in the sections correspond to the z-positions of the top and bottom XY slices (outlined in green and red, respectively). **b**, **d** Quantification of the z-position of the nuclei of dhBMECs and dhPCs relative to the glass surface after seeding dhPCs on dhBMECs (**b**) and dhBMECs on dhPCs (**d**). Data represent mean ± SD. For **b** and **d** at least 44 pairs of cells from at least 3 fields of view from at least 2 wells per seeding were quantified. **e** Collagen IV stain following seeding dhBMECs on dhPCs. The cross section is magnified (orange outline). White arrows in the cross-section indicate collagen IV above and below dhPCs. **f** Laminin stain following seeding dhBMECs on dhPCs. White arrows indicate laminin above and below dhPCs

To visually examine the result of sequential seeding of dhPCs and dhBMECs, we repeated the three transwell seeding conditions on coated glass. To distinguish each cell type, dhBMECs were either derived from GFP-expressing BC1 iPSCs or stained for Glut-1, and dhPCs were stained for calponin. Irrespective of the seeding order, dhPCs were predominantly localized between the dhBMEC monolayer and the coated glass substrate (Fig. 4a–d). This suggests that when seeded on a confluent dhBMEC monolayer, dhPCs migrate across the monolayer, disrupting cell–cell junctions. In order to examine whether directly co-cultured dhPCs could downregulate TJ expression in dhBMECs, we performed RT-qPCR on 2D co-cultures of dhBMECs and dhPCs but did not observe any significant differences in the gene expression of claudin-5 or occludin, further suggesting barrier disruption by physical means (Additional file 1: Fig. S5).

To examine the localization of basement membrane proteins in direct 2D co-culture of dhPCs and dhBMECs, we stained for collagen IV and laminin. Notably, abundant meshes of both proteins were observed between dhPCs and the surface, as well as between dhPCs and dhBMECs (Fig. 4e, f). While collagen IV is used to coat the surface to promote cell attachment, its localization in both layers, along with laminin, suggests secretion by one or both cell types and matches in vivo organization where pericytes are embedded within the basement membrane [1].

**Fig. 5:**
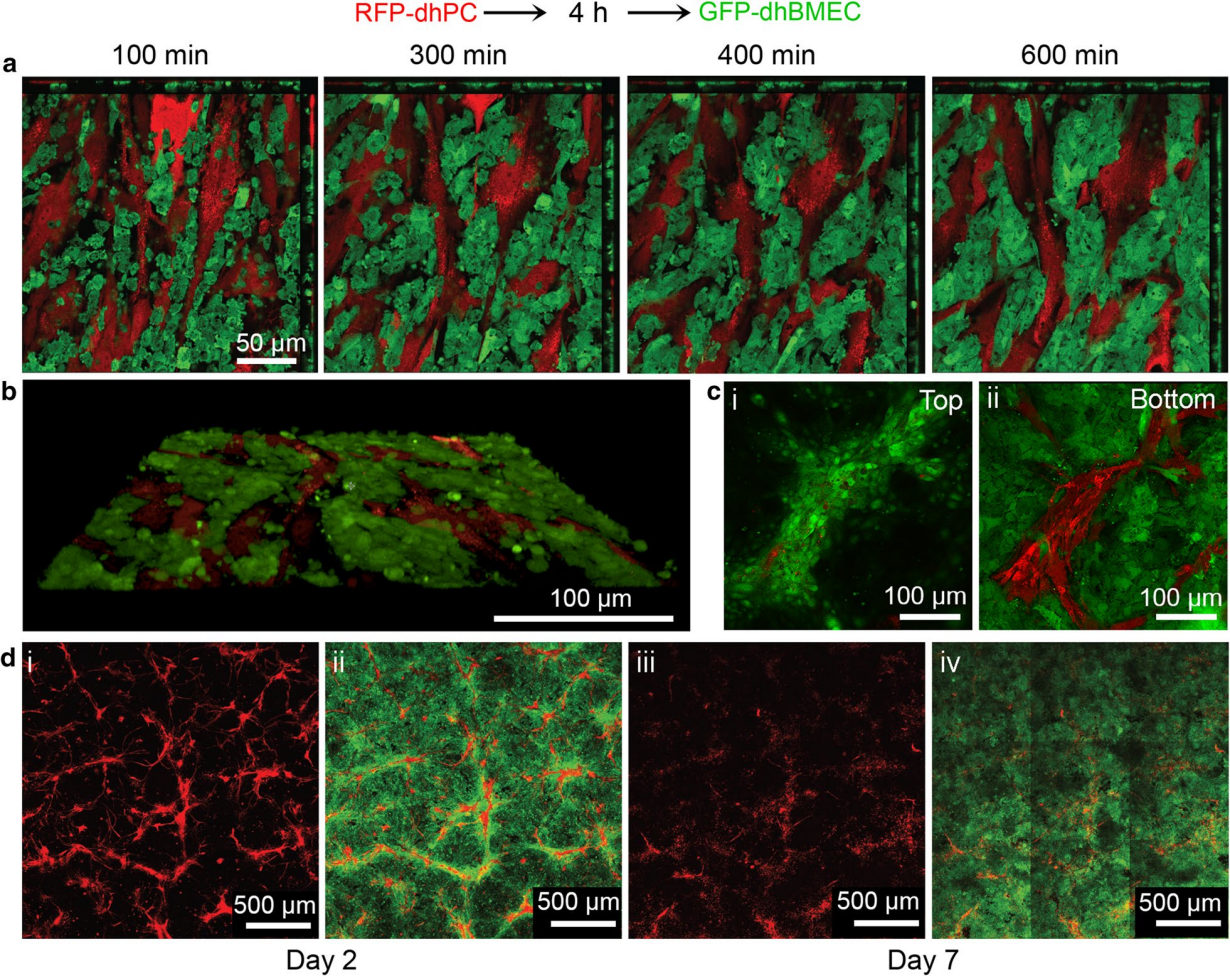
Confocal images of dhBMEC monolayer formation during 2D contact co-culture with dhPC. GFP-dhBMECs were seeded 4 h after RFP-dhPCs on collagen IV/fibronectin coated glass. **a** Images showing dhBMECs (green) and dhPCs (red) during the first 10 h after seeding dhBMECs. **b** 3D reconstruction after 10 h. **c** Confocal images 48 h after seeding dhBMECs, taken at the (i) upper and (ii) lower locations of the cell layer. **d** Epifluorescence imaging following seeding a mixture of dhPCs and dhBMECs after (i, ii) 2 days and (iii, iv) 7 days

To visualize the process of dhBMEC monolayer formation in the E-on-P seeding configuration in real time, we differentiated dhPCs from an RFP-expressing C12 iPSC line (see Additional file 1: Fig. S6) and seeded sub-confluent dhPCs on coated glass 4 h prior to seeding GFP-expressing dhBMECs (Fig. 5a, b). The dhBMECs did not initially adhere to the dhPCs, and hence a large portion of the surface was effectively blocked from dhBMEC attachment. During the first 10 h after seeding, small regions of confluent dhBMECs began to form. Within 2 days, dhBMECs appeared to migrate up over patches of dhPCs (Fig. 5c), resulting in the appearance of bright regions of dhBMECs immediately surrounding dhPCs (Fig. 5di, ii). Concurrent with proliferation and expansion of the dhBMEC regions, we observed a reduction in dhPC viability by day 7 (Fig. 5diii, iv), as characterized by the widespread shift of RFP-expressing cells into free RFP or RFP-containing vesicles, which appear to be readily taken up by dhBMECs. The origin of cell death may be due to restricted nutrient access resulting from dhBMEC overgrowth. From these results, we conclude that direct co-culture with dhPCs in 2D limits the ability of dhBMECs to form confluent monolayers.

### dhBMEC monolayers on dhPC-embedded hydrogels

**Fig. 6:**
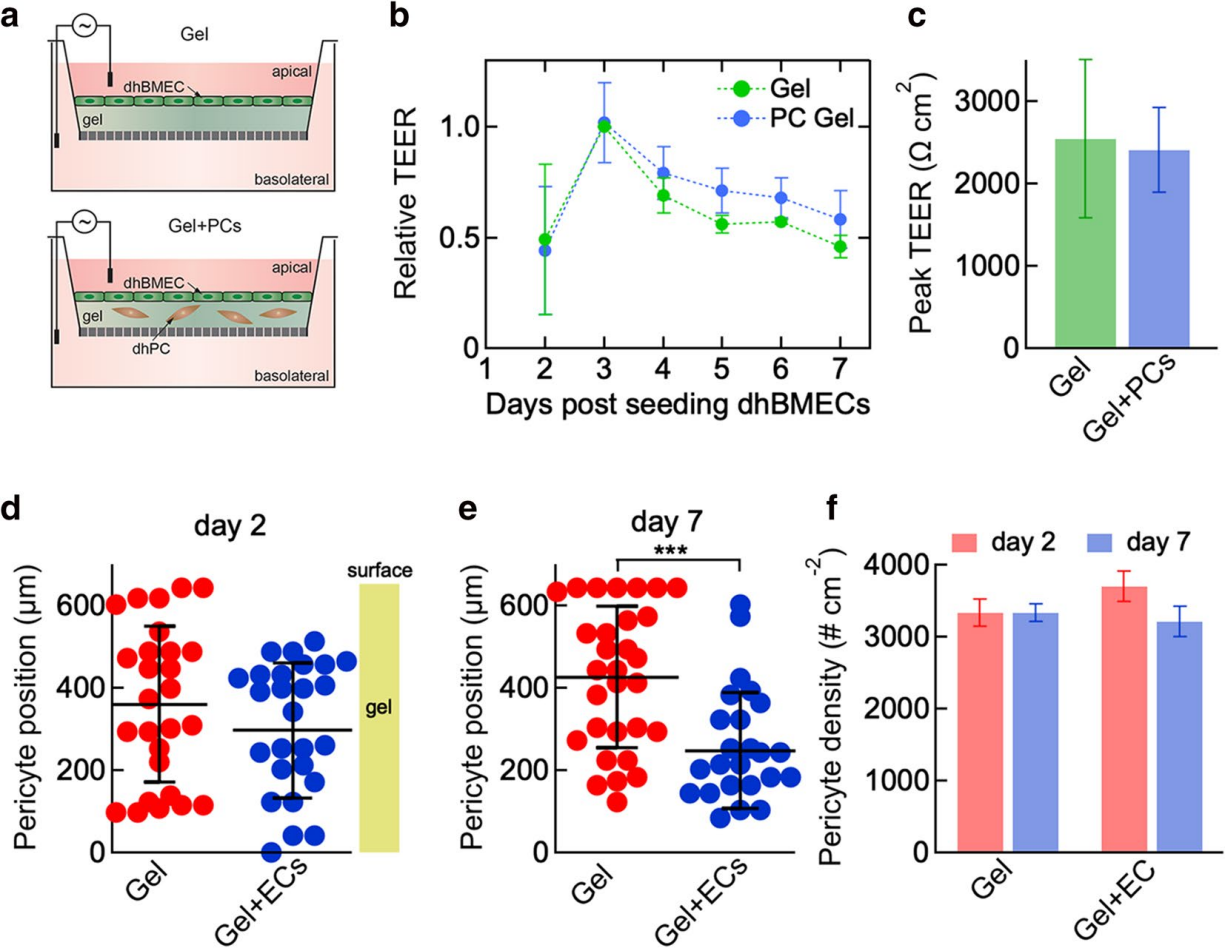
TEER, dhPC position, and dhPC density for dhBMEC monolayers on collagen I gels formed with encapsulated dhPCs. **a** Schematic illustration showing the 2.5D gel/transwell device. Experiments were performed with dhBMEC monolayers formed on 600 µm thick collagen gels (2.5 mg mL^−1^) with or without encapsulated dhPCs. **b** Time dependence of TEER values for dhBMECs on gels with and without dhPCs. TEER values have been normalized to the peak of the control (no dhPCs), such that each biological replicate of the control reaches a maximum relative TEER of 1.0 at its highest point. bc Peak TEER for dhBMECs on gels with and without dhPCs. In **b** and **c** data represent mean ± SEM for two biological replicates (differentiations) and at least two technical replicates (transwells). **d**, **e** Position of dhPCs in gels with or without a dhBMEC monolayer on day 2 (**d**) or day 7 (**e**). The distance is referenced to the bottom of the well. **f** dhPC density in gels with or without a dhBMEC monolayer at day 2 and day 7. Data in **d**–**f** represent mean ± SD. At least 26 cells were quantified per condition per time point pooled from two gel replicates. ***P < 0.001

To overcome the limitations of 2D co-culture, confluent monolayers of dhBMECs were formed on 2.5 mg mL^−1^ collagen I gels containing an intermediate concentration of dhPCs (1:13 dhPC:dhBMEC ratio) on a transwell insert (Fig. 6a). Note that the gel alone does not contribute to the measured TEER values (Additional file 1: Fig. S7). The gelation process was optimized to maintain dhPC viability during seeding, as verified by a calcein AM/propidium iodide live/dead assay (Additional file 1: Fig. S8). In this configuration, there was no significant difference in TEER for dhBMEC monolayers on collagen gels with or without embedded dhPCs (Fig. 6b). Furthermore, peak TEER values for these conditions were also comparable (Fig. 6c).

To assess how dhPCs respond to the presence of dhBMEC monolayers, the z-position of dhPCs in gels was determined from confocal images of gels with and without dhBMECs on top. After 2 days in gels without a dhBMEC monolayer, the dhPCs were distributed relatively uniformly in the gel from the surface to the well bottom (about 600 µm) (Fig. 6d). However, in gels with a dhBMEC monolayer, the dhPCs were excluded from the surface region near the dhBMECs and their average position was 60 µm deeper into the gel (Fig. 6d). After 7 days, this gap increased to 180 µm deeper, relative to gels without dhBMECs (Fig. 6e). The density of dhPCs was maintained relatively constant in either gel between day 2 and day 7 (Fig. 6f). Collectively, these results demonstrate the utility of a 2.5D dhBMEC/dhPC co-culture platform that permits dhBMEC monolayer formation, dhPC migration, and conventional TEER measurement.

### Tissue-engineered dhPC/dhBMEC microvessels

**Fig. 7:**
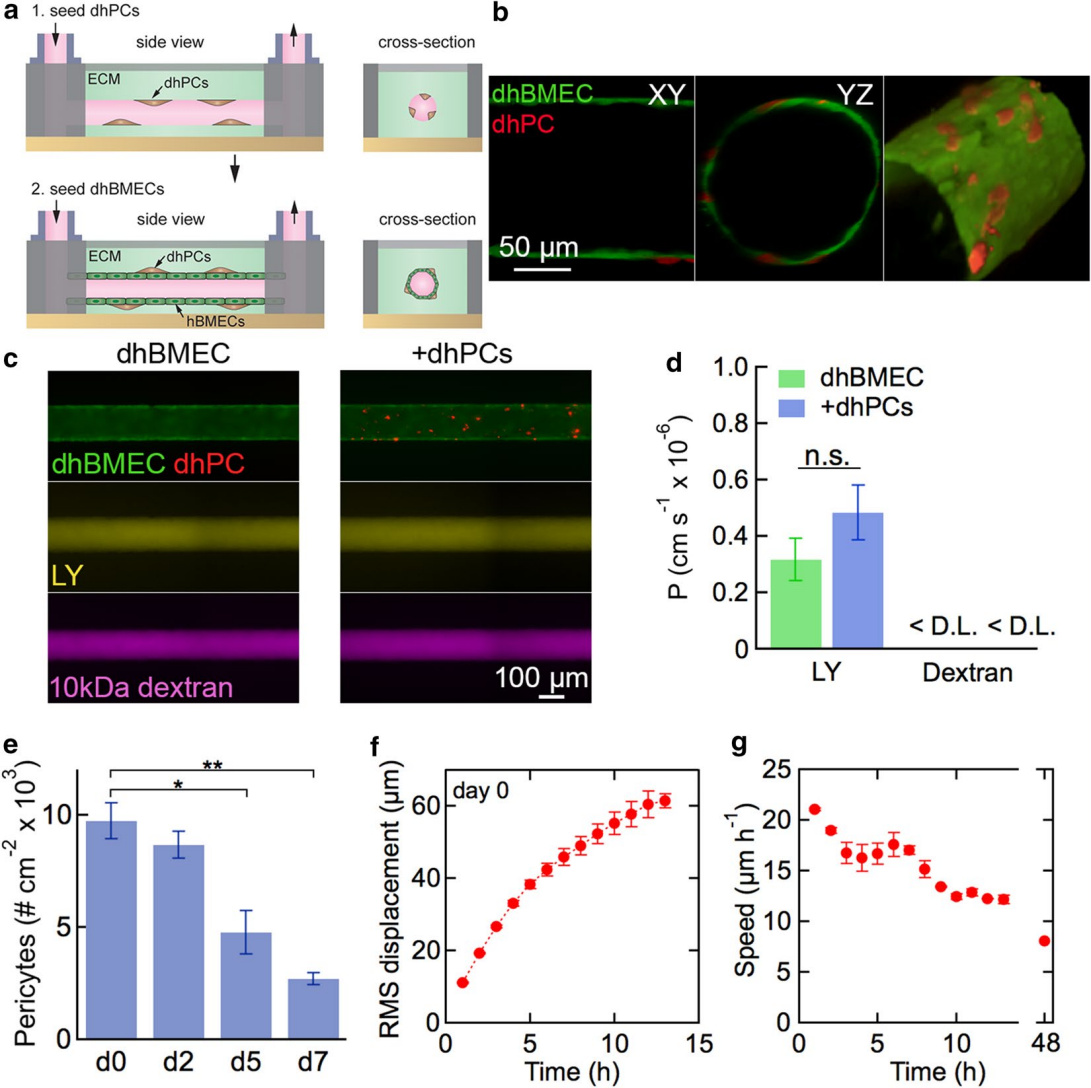
Tissue-engineered dhBMEC/dhPC microvessels. **a** Schematic illustration showing fabrication of microvessels with sequential seeding of dhPCs and dhBMECs in a cylindrical channel surrounded by collagen I. **b** Confocal slices of the XY and YZ planes, and a 3D reconstruction from confocal slices demonstrating dhPC (red) localization abluminal to dhBMECs (green), imaged on day 2 following seeding. **c** Fluorescence images of dhBMEC and dhBMEC/dhPC microvessels (+dhPC) (top) after 20 min of perfusion with: (middle) Lucifer yellow (LY) and (bottom) 10 kDa dextran. **d** Permeability of LY and 10 kDa dextran in dhBMEC microvessels with and without dhPCs on day 2. D.L.—detection limit. Bars represent mean ± SEM for three independent microvessels (N = 3). **e** Density of abluminal dhPCs over 7 days after seeding dhBMECs. Bars represent mean ± SEM (N = 2–4). **f** Root mean square (RMS) displacement of dhPCs along the lumen/matrix interface as a function of time immediately after seeding dhBMECs (day 0). Bars represent mean ± SEM (N = 2). **g** Average instantaneous speed of dhPCs along the lumen/matrix interface versus time. Bars represent mean ± SEM (N = 2). At least 65 cells were tracked per microvessel in **f** and **g**. *P < 0.05, **P < 0.01

To investigate dhPC/dhBMEC interactions in a platform that recapitulates shear stress and cylindrical geometry, we incorporated dhPCs into 3D tissue-engineered dhBMEC microvessels approximately 150 µm in diameter. Device fabrication and characterization have been reported elsewhere [27, 31, 42–44]. dhPCs were seeded into the channel that forms the microvessel 1 h prior to seeding dhBMECs (Fig. 7a). After seeding, microvessels were perfused with gravity-driven flow to provide approximately 4 dyn cm^−2^ shear stress, characteristic of post-capillary venules [45]. Confocal microscopy images show dhPCs located abluminally at the interface between the dhBMECs and the matrix (Fig. 7b). The dhBMECs form a confluent monolayer isolating the dhPCs from the microvessel lumen. Barrier function was assessed from simultaneous measurement of the permeability of Lucifer yellow (LY) and 10 kDa dextran, as reported previously (Fig. 7c) [27, 31]. LY is a small molecule (444 Da) commonly used to assess paracellular permeability of endothelial monolayers [2]. The permeability of LY in the microvessels was about 4 × 10^−7^ cm s^−1^, close to values reported previously for dhBMECs in a transwell assay [27], and there was no statistical difference for microvessels seeded with dhPCs (Fig. 7d). Furthermore, the presence of dhPCs did not result in any local focal leaks in the vicinity of the dhPCs (Fig. 7c). The permeability of 10 kDa dextran was below the detection limit for microvessels with and without dhPCs (Fig. 7d). Together, these data are consistent with our results in transwells where the presence of dhPCs in the basolateral chamber had no effect on TEER values for unstressed dhBMEC monolayers.

The density and motility of dhPCs were determined from live cell imaging of the microvessels. The density of dhPCs at the interface between the microvessel and the matrix remained constant for the first 2 days following seeding with dhBMECs. On day 2 when the permeability was measured, the dhPC density was about 1 × 10^4^ cm^−2^, corresponding to a dhPC:dhBMEC ratio of approximately 1:13. After 5–7 days, the number of dhPCs decreased, although there was no effect on the dhBMEC monolayer (Fig. 7e). Immediately after seeding dhBMECs, dhPCs migrate along the interface between the microvessel and the matrix with an RMS displacement of 60 µm (about 3 dhBMEC cells) over the first 12 h. The rate of change of the RMS displacement decreased with time as the dhPCs became spatially localized. The instantaneous cell speed was initially around 20 µm h^−1^, but decreased to about 5 µm h^−1^ after 2 days (Fig. 7f). Collectively, these results show that dhBMEC microvessels can be formed after seeding the matrix with dhPCs and that the dhPCs do not influence barrier function.

## Discussion

Developing physiological models of the BBB is extremely challenging due to the complex spatial architecture and the highly specialized nature of the microvascular endothelial cells that form the cerebrovasculature [46]. Incorporation of other cellular components of the neurovascular unit, such as pericytes, is also challenging since details of their role in barrier maintenance remain incomplete.

Depending on the region in the brain, pericytes arise from mesoderm or neural crest lineages [47, 48]. While there are an increasing number of new protocols for differentiation of pericyte-like cells, some of which proceed through neural crest intermediates [49, 50], for this study we selected a well-established differentiation for pericyte-like cells from a mesoderm lineage, which has been previously characterized, shown to support 3D vascular networks, and distinguished from vascular smooth muscle cells (VSMCs) [29, 30, 51]. Although neural crest-derived pericytes could improve barrier function (e.g. increase TEER) in non-stressed dhBMECs compared to mesoderm-derived pericytes, a recent comparison of pericyte-like cells derived from either neural crest or mesoderm showed no differences in their abilities to support 3D vascular networks and modulate TEER [50].

In this study, we employed established hiPSC differentiation protocols for dhBMECs and dhPCs based on their validated phenotype and systematically evaluated the effect of co-culture of dhPCs on paracellular transport across dhBMECs in multiple configurations. Note that although transcellular transport pathways may also be affected by pericyte co-culture [15, 16], they were not explicitly examined in this study.

Many studies with immortalized or primary BMECs in transwells have shown that indirectly co-cultured pericytes or astrocytes can increase TEER values, however, these values are usually well below the range thought to be physiological (1500–8000 Ω cm^2^) [20]. Here we show no influence of dhPCs on the TEER of dhBMEC monolayers when seeded in the basolateral chamber. However, we find that dhPCs can induce recovery of TEER for stressed dhBMEC monolayers. Other studies have shown mixed results regarding the effect of pericytes or other supporting cell types on TEER of dhBMEC monolayers [20, 33, 36, 41, 52–54]. These results support the hypothesis that optimal dhBMEC monolayers do not require other cell types to establish physiological barrier function, but that barrier function can be partially or fully rescued in stressed monolayers through secretion of soluble factors.

Direct co-culture of dhPCs with dhBMECs on the apical side of a transwell insert resulted in a decrease in TEER irrespective of the dhPC:dhBMEC ratio or the seeding order. Imaging co-cultures on glass slides show that dhPCs outcompete dhBMECs for the glass surface such that dhPCs will migrate through dhBMECs if seeded on top, and will force dhBMECs to overgrow dhPC clusters if the dhBMECs are seeded after the dhPCs. These results suggest that 2D models are not capable of recapitulating the spatial arrangement of pericytes and brain microvascular endothelial cells in co-culture.

Culture of dhBMECs on gels containing dhPCs showed no change in TEER values compared to controls with no dhPCs. This geometry resulted in a more physiological spatial arrangement of cells with the dhBMEC monolayer formed on the gel surface and with dhPCs able to migrate through the gel. While brain microvascular endothelial cells are known to recruit pericytes during development [15–17], we observed that dhPCs migrated away from dhBMEC monolayers formed on top of the gels. This effect could be due to nutrient depletion in the vicinity of the dhBMEC monolayer or from cues associated with vascular remodeling, as occurs during early stage cerebrovascular angiogenesis [13, 14].

In dhPC/dhBMEC microvessels, we recapitulated the correct spatial arrangement with sparse dhPCs located at the interface between the endothelium and the surrounding matrix. With the dhPCs seeded on the curved matrix surface, dhBMECs were able to form a confluent monolayer without discontinuities. The permeability of LY in dhBMEC microvessels was the same with and without dhPCs. The permeability of 10 kDa dextran was below the detection limit in both cases. Therefore, the presence of dhPCs in transwells or in microvessels had no effect on barrier function of healthy dhBMEC monolayers. In contrast, co-cultured human bone marrow stromal cells reduced the permeability of 10 kDa dextran 10–20-fold in microvessels formed from human umbilical vein endothelial cells [55], suggesting that stromal cells may play an important role in regulating barrier function in other tissues.

Recent two photon microscopy studies in the mouse cortex demonstrate that isolated capillary pericytes show negligible migration over 30 days [7, 8, 56], suggesting that pericytes are stationary in the healthy BBB. This may thus constitute an important criterion for recapitulating physiological pericytes in vitro. dhPC motility at the interface between the dhBMECs and the matrix was relatively low upon seeding (RMS displacement of ≈ 60 µm over the first 12 h) and dhPC speed decreased significantly with time (dropping 60% by day 2) suggesting dhPC could be approaching a non-motile state in the 3D microvessel model.

## Conclusions

Here we report co-culture of iPSC-derived pericytes and BMEC in three configurations: 2D co-culture in a transwell, 2.5D culture with dhBMEC monolayers on a dhPC-embedded gel, and 3D co-culture of dhPCs in tissue-engineered microvessels. Depending on the configuration, seeding sequence, and concentration, dhPCs either have no effect on barrier function or reduce barrier function of healthy dhBMEC monolayers. These results support the hypothesis that pericytes are not essential for establishing barrier function in healthy dhBMEC monolayers, and indeed in some configurations, can prevent barrier establishment. However, dhPCs are able to rescue barrier function in stressed dhBMEC monolayers through the secretion of soluble factors.

## Additional file

**Additional file 1: Table S1.** Antibodies used in this study. **Figure S1.** Comparison of dhPC to primary human pericytes. **Figure S2.** Effect of ROCK inhibitor on dhBMEC seeding. **Figure S3.** Barrier function of dhBMEC monolayers on transwells in non-contact co-culture with dhPCs or dhPC-conditioned media. **Figure S4.** Effect of conditioned media on dhBMEC barrier function and TJs. **Figure S5.** RT-qPCR analysis of tight junction gene expression in 2D direct contact co-cultures. **Figure S6.** Immunofluorescent staining of dhPC derived from the C12-RFP line for established pericyte and mural cell markers. **Figure S7.** Baseline TEER for transwell membrane, transwell membrane with collagen I gel, or transwell membrane with PC-embedded collagen I gel. **Figure S8.** Viability of dhPC embedded in collagen-1 gels.

## Data Availability

Not applicable.

## Abbreviations

BBB: blood–brain barrier
ECs: endothelial cells
CNS: central nervous system
TJs: tight junctions
hiPSCs: human induced pluripotent stem cells
NVU: neurovascular unit
BMECs: brain microvascular endothelial cells
BM: basement membrane
ECM: extracellular matrix
TEER: transendothelial electrical resistance
