# Supplementary material for: Role of iPSC-derived pericytes on barrier function of iPSC-derived brain microvascular endothelial cells in 2D and 3D
Source: Fluids Barriers CNS. 2019 Jun 6;16:15. doi: 10.1186/s12987-019-0136-7 (PMC6551886; doi:10.1186/s12987-019-0136-7)
Supplement: Supplementary file 1 — Additional file 1: Table S1. Antibodies used in this study. Figure S1. Comparison of dhPC to primary human pericytes. Figure S2. Effect of ROCK inhibitor on dhBMEC seeding. Figure S3. Barrier function of dhBMEC monolayers on transwells in non-contact co-culture with dhPCs or dhPC-conditioned media. Figure S4. Effect of conditioned media on dhBMEC barrier function and TJs. Figure S5. RT-qPCR analysis of tight junction gene expression in 2D direct contact co-cultures. Figure S6. Immunofluorescent staining of dhPC derived from the C12-RFP line for established pericyte and mural cell markers. Figure S7. Baseline TEER for transwell membrane, transwell membrane with collagen I gel, or transwell membrane with PC-embedded collagen I gel. Figure S8. Viability of dhPC embedded in collagen-1 gels. [file 12987_2019_136_MOESM1_ESM.pdf]

Role of iPSC-derived pericytes on barrier function of iPSC-derived brain microvascular endothelial cells in 2D and 3D

John J. Jamieson[1,2], Raleigh M. Linville[2,3], Yuan Yuan Ding[1,2], Sharon Gerecht[1,2,3,4,*], and Peter C. Searson[2,4,*]

ADDITIONAL INFORMATION

Table S1. Antibodies used in this study
Figure S1. Comparison of dhPC to primary human pericytes.
Figure S2. Effect of ROCK inhibitor on dhBMEC seeding.
Figure S3. Barrier function of dhBMEC monolayers on transwells in non-contact co-culture with dhPCs or dhPC-conditioned media.
Figure S4. Effect of conditioned media on dhBMEC barrier function and TJs
Figure S5. RT-qPCR analysis of tight junction gene expression in 2D direct contact co-cultures.
Figure S6. Immunofluorescent staining of dhPC derived from the C12-RFP line for established pericyte and mural cell markers.
Figure S7. Baseline TEER for transwell membrane, transwell membrane with collagen I gel, or transwell membrane with PC-embedded collagen I gel.
Figure S8. Viability of dhPC embedded in collagen-1 gels.

Antibody	Source	Catalog #	Application	Host Species & Reactivity	Dilution
αSMA	Dako	M0851	ICC	Mouse anti-human	1:100
Calponin	Santa Cruz	sc-58707	ICC	Mouse anti-human	1:100
CD31	Dako	M0823	ICC	Mouse anti-human	1:100
CD73-PE	R&D Systems	fab5795p	FC	Mouse anti-human	1:10
CD105-PE	R&D Systems	fab10971p	FC	Mouse anti-human	1:10
Claudin-5	Thermo Fisher	35-2500	ICC	Mouse anti-human	1:100
Collagen IV	abcam	ab6586	ICC	Rabbit anti-human	1:200
Glut-1	Santa Cruz	sc-1605	ICC	Goat anti-human	1:100
IgG-PE	BD Biosciences	555749	FC	Mouse IgG Isotype Control	1:10
Laminin	abcam	ab11575	ICC	Rabbit anti-human	1:200
NG2	Santa Cruz	sc-166251	ICC	Mouse anti-human	1:100
Occludin	Thermo Fisher	71-1500	ICC	Rabbit anti-human	1:100
PDGFRβ	Santa Cruz	sc-432	ICC	Rabbit anti-human	1:100
PDGFRβ-PE	BD Biosciences	558820	FC	Mouse anti-human	1:10
VECad	Santa Cruz	sc-9989	ICC	Mouse anti-human	1:100
VECad-PE	BD Biosciences	560410	FC	Mouse anti-human	1:10
ZO-1	Thermo Fisher	61-7300	ICC	Rabbit anti-human	1:100
Alexa Fluor 488	Thermo Fisher	a11008	ICC	Goat anti-rabbit	1:1000
Alexa Fluor 488	Thermo Fisher	a11055	ICC	Donkey anti-goat	1:1000
Alexa Fluor 488	Thermo Fisher	a11029	ICC	Goat anti-mouse	1:1000
Alexa Fluor 546	Thermo Fisher	a11035	ICC	Goat anti-rabbit	1:1000
Alexa Fluor 546	Thermo Fisher	a10036	ICC	Donkey anti-mouse	1:1000
Alexa Fluor 546-Phalloidin	Thermo Fisher	a22283	ICC	f-actin	1:1000

Table S1. Antibodies used in this study. ICC = Immunocytochemistry; FC = Flow cytometry

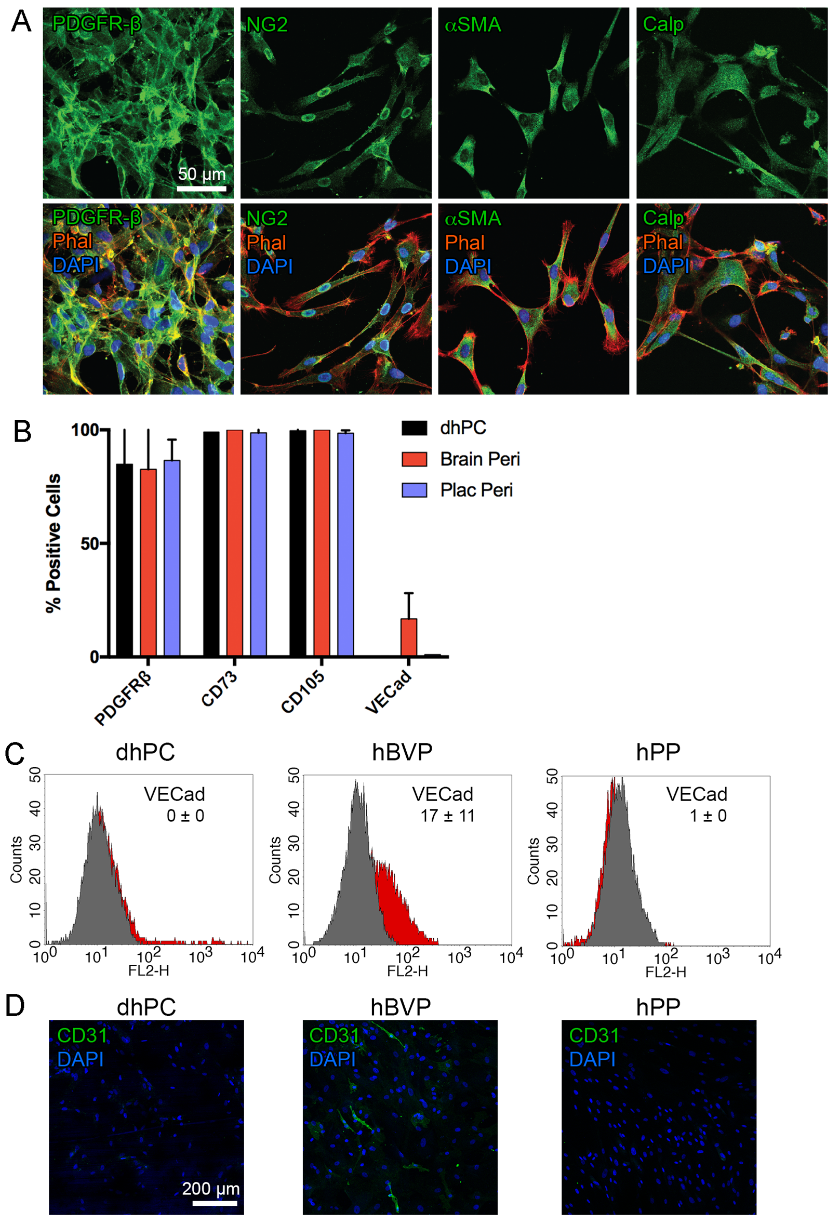

Figure S1. Comparison of dhPCs and primary human pericytes. (A) Immunofluorescence staining of primary human brain vascular pericytes for established pericyte and mural cell markers. (B) Flow cytometry comparison of dhPCs, human brain vascular pericytes, and human placental pericytes. Data represent mean ± SD of at least three biological replicates. *P < 0.05. (C) Representative live-cell flow cytometry histograms of dhPCs for VECad. The percentages indicate mean ± SD of at least three biological replicates. (D) Immunofluorescence staining for CD31 expression for dhPCs, primary brain pericytes, and primary placental pericytes.

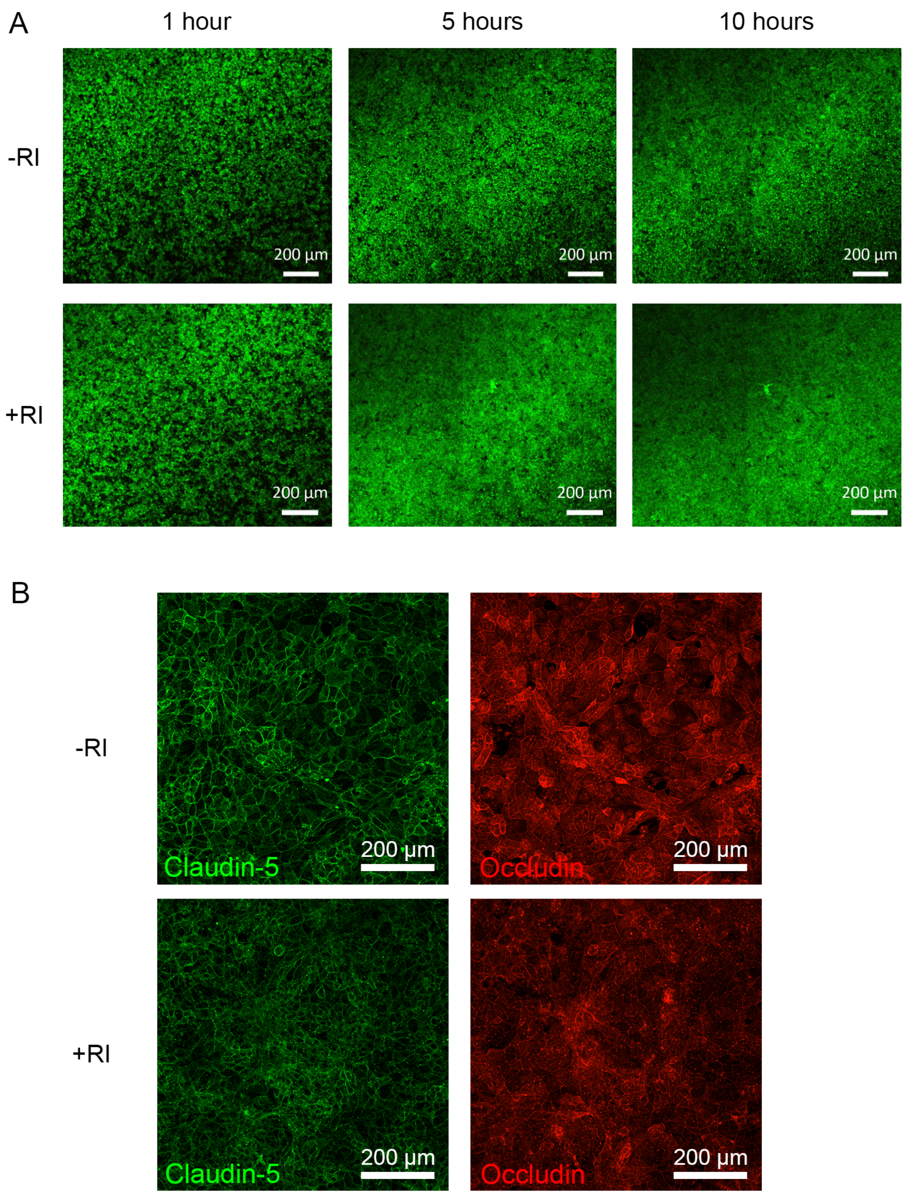

Figure S2. Effect of ROCK inhibitor on dhBMEC seeding. (A) GFP-expressing dhBMECs seeded with (+RI) or without (-RI) ROCK inhibitor. Images are shown at 1 hour, 5 hours, and 10 hours post seeding. (B) Immunofluorescence staining of dhBMECs for claudin-5 and occludin two days after seeding on glass coated with collagen IV and fibronectin. Media was replaced 1 day after seeding to remove ROCK inhibitor and any unattached cells.

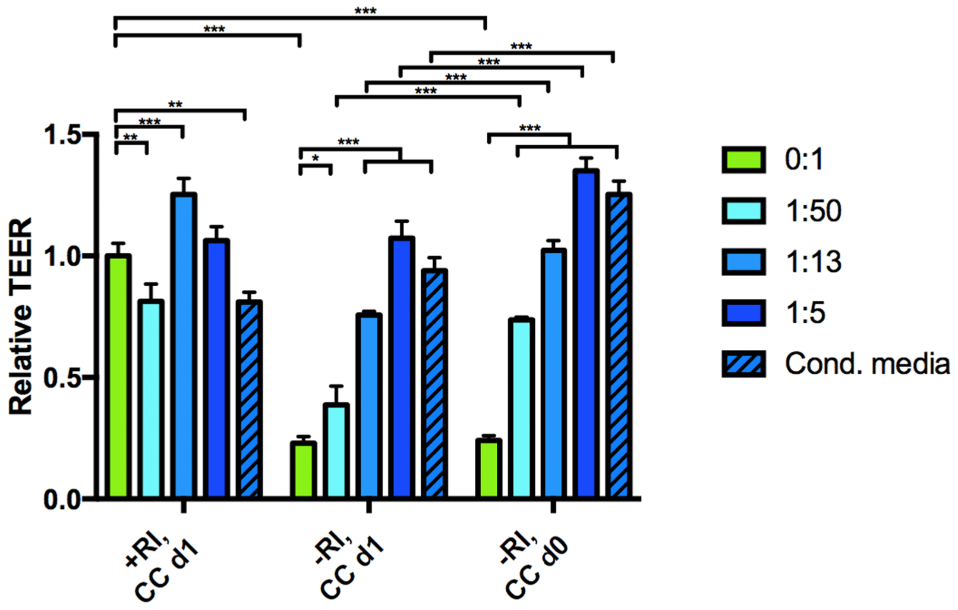

Figure S3. Barrier function of dhBMEC monolayers on transwells in non-contact co-culture with dhPCs or dhPC-conditioned media. +RI denotes ROCK inhibitor (Y27632) was included during the first 24 hours of subculture (from day 0 to day 1). CC d1 refers to the initiation of co-culture on day 1, while CC d0 refers to the initiation of co-culture on day 0 (dhPC present during dhBMEC seeding). Peak TEER values were normalized to the control (0:1 dhPC:dhBMEC, +RI). Data represent mean ± SD of three replicate transwells. * P <0.05, ** P < 0.01, *** P < 0.001.

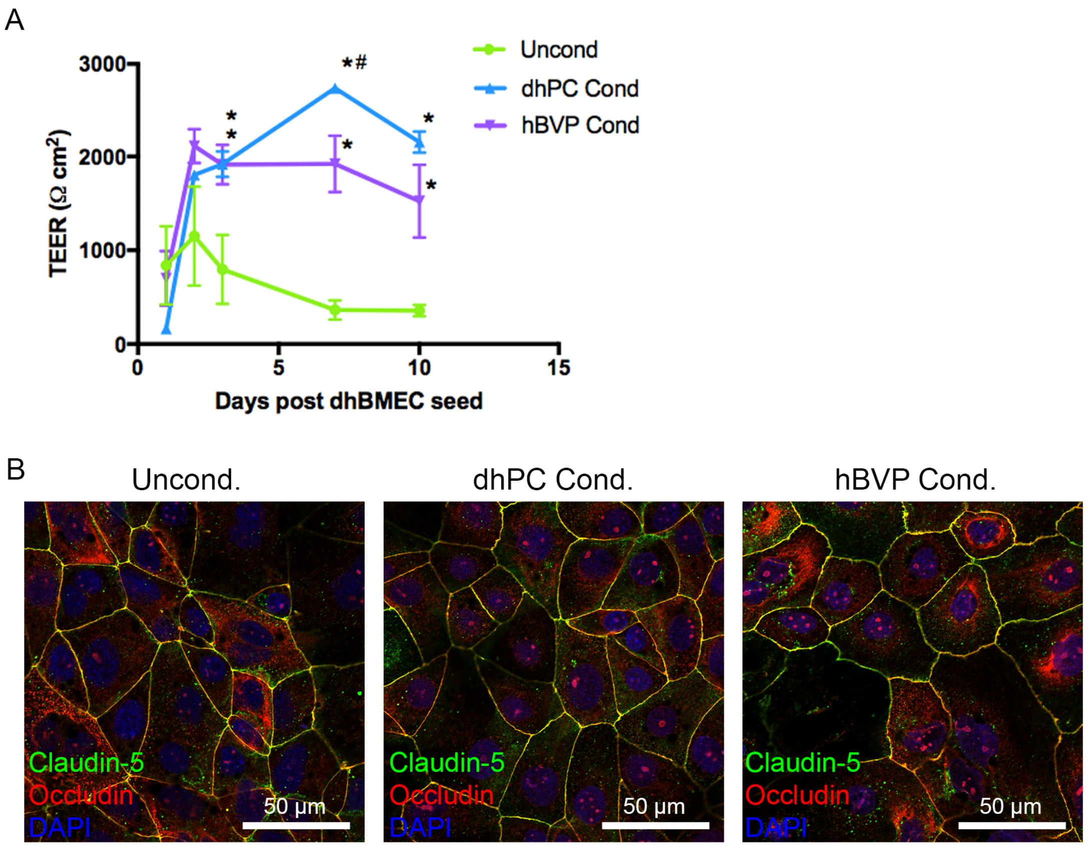

Figure S4. Effect of conditioned media on dhBMEC barrier function and TJs. (A) TEER of dhBMEC seeded in unconditioned, dhPC-conditioned, or hBVP-conditioned dhBMEC media. '*' denotes statistically significant ($P < 0.05$) difference from unconditioned dhBMEC media. '#' denotes statistically significant ($P < 0.05$) difference from hBVP-conditioned dhBMEC media. Experiments were performed with two biological replicates (independent dhBMEC differentiations) and two technical replicates (transwells for each differentiation). (B) Immunofluorescence staining of dhBMEC for claudin-5, occludin, and DAPI two days after seeding in unconditioned, dhPC-conditioned, or hBVP-conditioned dhBMEC medias on glass coated with collagen IV and fibronectin. Medias were replaced 1 day after seeding to remove any unattached cells.

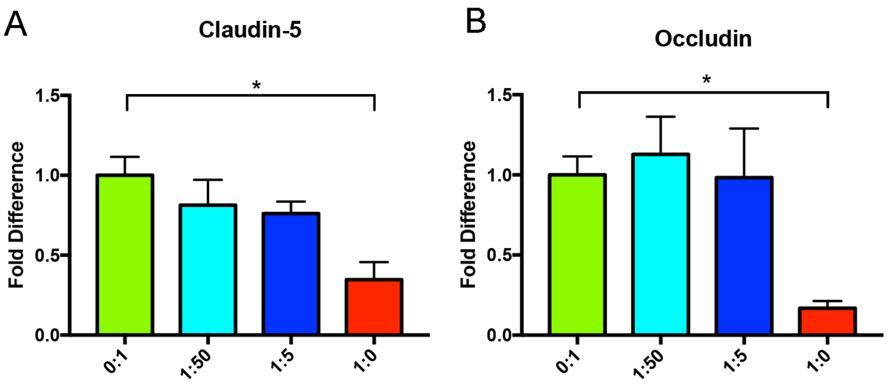

Figure S5. RT-qPCR analysis of tight junction gene expression in 2D direct contact co-cultures. (A) Claudin-5 gene expression in dhBMECs and dhPCs following co-culture with different dhPC:dhBMEC seeding ratios. (B) Occludin gene expression in dhBMECs and dhPCs following co-culture with different dhPC:dhBMEC seeding ratios. Data represent mean ± SEM of three biological replicates (independent differentiations). * $P < 0.05$.

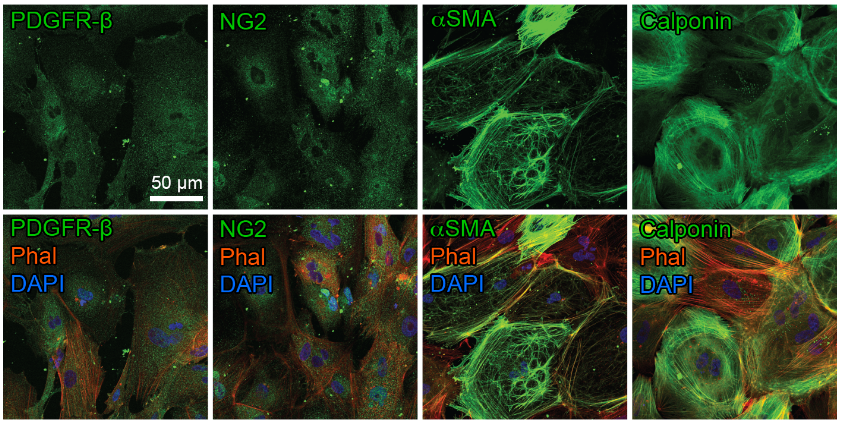

Figure S6. Immunofluorescence stains of dhPCs (derived from the C12-RFP) line for established pericyte and mural cell markers.

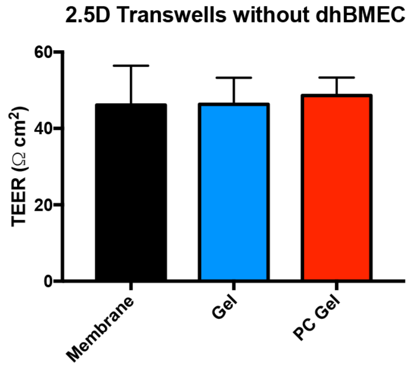

Figure S7. Baseline TEER (no dhBMECs) for a transwell membrane, transwell membrane with collagen I gel, or transwell membrane with PCs embedded in a collagen I gel. Data represent mean ± SD of six replicate transwells.

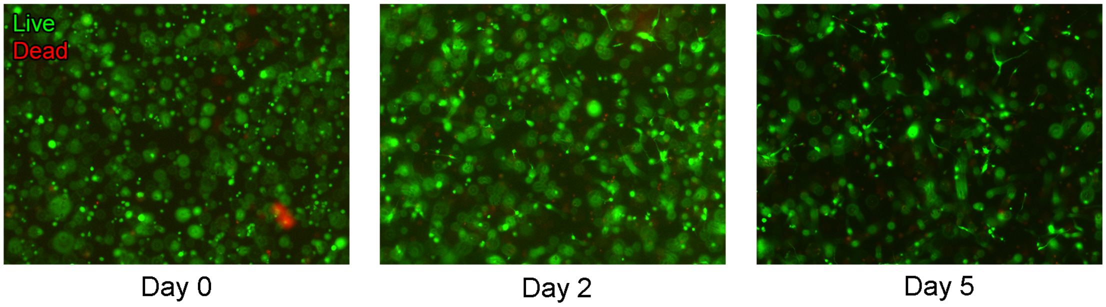

Figure S8. Viability of dhPC embedded in collagen-1 gels. (Green) live cells (calcein-AM). (Red) dead cells (propidium iodide).
